# *Boamente*: A Natural Language Processing-Based Digital Phenotyping Tool for Smart Monitoring of Suicidal Ideation

**DOI:** 10.3390/healthcare10040698

**Published:** 2022-04-08

**Authors:** Evandro J. S. Diniz, José E. Fontenele, Adonias C. de Oliveira, Victor H. Bastos, Silmar Teixeira, Ricardo L. Rabêlo, Dario B. Calçada, Renato M. dos Santos, Ana K. de Oliveira, Ariel S. Teles

**Affiliations:** 1 Federal Institute of Maranhão, Araioses 65570-000, Brazil; evandro.diniz@ifma.edu.br; 2 Technological Neuro Innovation Laboratory, Federal University of Delta do Parnaíba, Parnaíba 64202-020, Brazil; vetophp@gmail.com (J.E.F.); adonias.ifce@gmail.com (A.C.d.O.); victorhugobastos@ufpi.edu.br (V.H.B.); silmarteixeira@ufpi.edu.br (S.T.); renatomendes@ufpi.edu.br (R.M.d.S.); 3 Department of Electrical Engineering, Federal University of Piauí, Teresina 64049-550, Brazil; ricardoalr@ufpi.edu.br (R.L.R.); anakarla_deoliveira@yahoo.com.br (A.K.d.O.); 4 Department of Computer Science, State University of Piauí, Parnaíba 64202-220, Brazil; dariobcalcada@frn.uespi.br

**Keywords:** artificial intelligence, deep learning, eHealth, mental health, mobile application, natural language processing, suicide

## Abstract

People at risk of suicide tend to be isolated and cannot share their thoughts. For this reason, suicidal ideation monitoring becomes a hard task. Therefore, people at risk of suicide need to be monitored in a manner capable of identifying if and when they have a suicidal ideation, enabling professionals to perform timely interventions. This study aimed to develop the *Boamente* tool, a solution that collects textual data from users’ smartphones and identifies the existence of suicidal ideation. The solution has a virtual keyboard mobile application that passively collects user texts and sends them to a web platform to be processed. The platform classifies texts using natural language processing and a deep learning model to recognize suicidal ideation, and the results are presented to mental health professionals in dashboards. Text classification for sentiment analysis was implemented with different machine/deep learning algorithms. A validation study was conducted to identify the model with the best performance results. The BERTimbau Large model performed better, reaching a recall of 0.953 (accuracy: 0.955; precision: 0.961; F-score: 0.954; AUC: 0.954). The proposed tool demonstrated an ability to identify suicidal ideation from user texts, which enabled it to be experimented with in studies with professionals and their patients.

## 1. Introduction

### 1.1. Background

Suicide is one of the main causes of death in the world [1]. In 2019, Brazil was among ten countries where the most suicides occurred in the world, and the second among countries of the Americas, with 14,540 suicide cases [2]. According to the World Health Organization (WHO), 703,000 people committed suicide in 2019 in the world. As an aggravating factor, the current COVID-19 pandemic has changed people’s well-being and mental health due to different events, such as deaths, social isolation, and job closures, which can also cause an increase in the number of people at risk of suicide [3,4].

Several factors can influence individuals to make the decision to end their lives (for example, emotional pain, marital problems, and biological, genetic, psychological, social, cultural, financial, and environmental factors) [5,6,7]. According to the WHO, when people are mentally healthy, they are able to be productive, contribute to the community, and recover from the stress they experience daily [1]. In contrast, mental disorders can negatively impact people’s lives, in addition to affecting relationships with friends, family, and health systems. Anyone can have suicidal ideation [8].

To prevent suicide, there has been rapid growth in the development and use of digital technologies [9], such as mobile applications [10,11], which can identify, monitor, and support individuals at risk. In particular, mobile applications for digital phenotyping aim at collecting information to objectively contribute to the identification of symptoms and behaviors of interest to mental health professionals (for example, psychologists and psychiatrists) [12,13]. According to Torous et al. [14], the term “digital phenotyping” refers to a “moment-by-moment quantification of the individual-level human phenotype in-situ using data from smartphones and other personal digital devices”. Digital phenotyping mobile applications use people’s interactions with smartphone applications in everyday environments to facilitate remote monitoring of their behaviors and habits, requiring little or no direct interaction for data collection.

Usually, people at risk of suicide tend to be isolated and cannot share their suicidal thoughts with their family, friends, or even mental health professionals [15]. At the same time, people may express their emotions, thoughts, and feelings in a variety of ways, including through text messages on social media (for example, Twitter, Facebook, Instagram, and Reddit) [16]. These texts, obtained from online social media, may be defined as non-clinical texts [17], as they are not annotated by health professionals. Non-clinical texts can be obtained from different sources, but social media can produce large quantities available at any time. Such a characteristic (the high availability at any time) enables non-clinical texts to be explored in studies that use machine/deep learning (ML/DL) and natural language processing (NLP) techniques to identify suicidal ideation [18]. Such techniques have demonstrated their potential to perform different tasks in the healthcare field [19,20].

### 1.2. Related Work

Suicide is an intriguing form of human death, and its motivations are complex [21]. Therefore, the timely identification of an individual at risk of suicide is a hard task. For this reason, different studies have taken advantage of information and communication technologies (ICT), such as ML/DL techniques [22,23,24] and mobile applications [10,25,26], to identify suicidal patterns and behaviors. Such studies seek to propose computer solutions enabled for the early identification of people at risk of suicide. Thus, solutions are proposed to prevent suicide from happening.

Most mobile applications for suicide prevention provide features for ecological momentary assessment (EMA) [27] and ecological momentary intervention (EMI) [28], such as *emma* [29], and coping tools, such as *CALMA* [30]. There are a few digital phenotyping applications for suicide prevention and, specifically, solutions focused on detecting suicidal ideation. *Strength Within Me* [31,32] is a digital phenotyping mobile application developed to sense data that are useful for predicting suicidality. It collects contextual information, usually gathered from smartphone sensors, such as sleep behavior, mood, and steps, to be correlated with user answers obtained from a suicide severity rating scale. The collected data are used as inputs to test ML models for predicting suicide risk. Studies using this digital phenotyping application [31,32] demonstrated its feasibility to detect risk of suicidality.

Another proposed digital phenotyping mobile application for monitoring suicidal ideation is *SIMON* [33]. This solution is composed of two parts: *SIMON-SELF*, which is an EMA application that uses a conversational agent (a chatbot) to request self-reports from users; and *SIMON-SENSE*, a sensing application used to passively collect contextual data from the user’s smartphone (for example, data produced by an accelerometer, GPS, Bluetooth, Wi-Fi) and identify situations of interest (for example, physical activity, location, and social connectedness). Collected data will be used as inputs to develop ML models for predicting suicidal ideation and psychiatric hospital re-admission.

Studies focusing on developing ML/DL models may use non-clinical texts to identify harmful content related to suicide. Burnap et al. [34] used non-clinical texts related to suicide to train several ML algorithms. This study aimed at classifying texts relating to suicide on Twitter. The study motivation is based on the fact that suicide-related posts can represent a risk to the users of online social networks, who could encourage them to hurt themselves. Classifiers were trained to distinguish between suicidal ideation and other suicide-related content (for example, suicide reports, memorials, campaigning, and support).

Psychiatric stressors (see [35] for definition) related to suicide were detected by Du et al. in [36]. For this purpose, the authors used user posts (non-clinical texts) obtained from Twitter and a convolutional neural network (CNN) to classify them into positive (that is, related to suicide or suicide ideation) and negative (that is, unrelated to suicide or suicide ideation) classes. Next, psychiatric stressors were annotated in the tweets labeled as positive, and a recurrent neural network (RNN) was used to extract stressors from positive tweets. Models created using different ML/DL algorithms were compared to identify the best one. This study achieved promising results in the process of identifying psychiatric stressors.

In the work by Ophir et al. [37], two deep neural network models using the Facebook posts of users to predict suicide risk were developed. The first model was able to predict suicide risk from posts. The second one was focused on predicting a hierarchical combination of multiple factors (for example, personality traits, psychosocial risks, and psychiatric disorders) to mediate the link between Facebook posts and suicide risk.

Most of the works have developed models using the English language as input. Carvalho et al. [38] started the study for suicidal ideation detection using texts written in Brazilian Portuguese (PT-BR). This work used texts obtained from Twitter to develop and compare three different ML/DL models. Posts were labeled using two approaches: three classes (safe to ignore, possibly worrying, and strongly worrying) and two classes (safe to ignore and possibly worrying). Results demonstrated that the bidirectional encoder representations from transformers (BERT) model [39] obtained the best performance in the two approaches; we have considered the models developed in Carvalho et al.’s study to compare with our model.

### 1.3. Rationale for Study

Different from the previous works, we have put together, in a smart mHealth solution, the strengths of artificial intelligence (AI) by using a state-of-the-art DL model, NLP techniques, and digital phenotyping, and by passively collecting texts typed by users to enable health professionals to monitor the suicide ideation of their patients. Our study aimed to develop the *Boamente* (the Portuguese word for “good mind”) tool, a virtual keyboard typing-based mobile tool for the digital phenotyping of mental health, to remotely detect and monitor the suicidal ideation of users. Potential users of the proposed solution are patients undergoing treatment with mental health professionals.

Our mHealth tool seeks to identify suicidal ideation in people at risk of suicide, thus preventing the occurrence of suicide in fact. For this purpose, *Boamente* passively collects user texts using a custom virtual keyboard application, and sends them to a web platform, which classifies them using NLP and DL techniques into two classes, negative and positive, of suicidal ideation. Next, the texts are discarded, and classification results are stored in a database. Finally, the web application displays results in dashboards to allow mental health professionals to monitor the suicide ideation of patients.

In summary, this study aimed to develop:

1. A BERT-based deep learning model for identifying the presence of suicidal ideation in non-clinical texts written in Brazilian Portuguese;
2. A digital phenotyping tool proposed to allow mental health professionals to monitor suicidal ideation of patients and, as a consequence, prevent suicide.

The remainder of this paper is organized as follows. Section 2 describes the methodology used to develop and evaluate the proposed solution, while Section 3 shows the results. In Section 4, we discuss the results, also providing plans for future work. Finally, Section 5 concludes the paper.

## 2. Methodology

### 2.1. Overview

A co-design method [40] was used to develop the *Boamente* solution, in which continuous feedback from psychologists was collected to contribute to the system’s development. Firstly, we developed an Android virtual keyboard able to passively collect user texts and send them to a web service. We then developed a web platform composed of a service to receive texts from keyboard applications, a component with the DL model deployed, and an application for data visualization. The latter is a web application with dashboards to display text analysis results to mental health professionals. Finally, we developed ML/DL models associated with NLP techniques to classify texts according to the presence (class positive) or absence (class negative) of suicidal ideation. Next, we present the result of a co-design with psychologists of the *Boamente* system and the methods used in the process of development and validation of the DL model for suicide ideation detection.

### 2.2. The Boamente System

**Figure 1:**
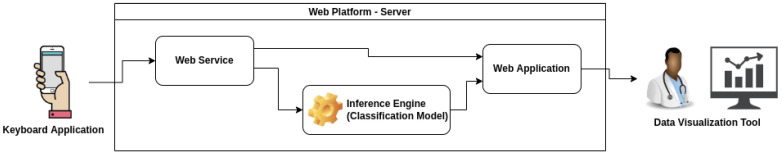
*Boamente* system overview.

Figure 1 depicts an overview of the *Boamente* solution. A virtual keyboard application was developed natively in Java language for Android OS using the integrated development environment (IDE) Android Studio. The application can replace Google’s default keyboard (Gboard [41]) on the user’s smartphone. The *Boamente* virtual keyboard was developed to passively capture texts typed by users, as Gboard is unable to provide such functionality. As potential users of the proposed solution are patients undergoing treatment, the application replacement should be recommended by their mental health professionals. Therefore, users (that is, patients) should install and use the *Boamente* virtual keyboard following medical advice.

The *Boamente* keyboard application captures every character typed by the user, but created texts are sent to the web service only if they have 2 (two) or more words. Along with the sentence, the creation timestamp of each sentence and a universally unique identifier (UUID) are also sent. The UUID is created and stored at the time of installation of the *Boamente* keyboard to identify users in the web application. The UUID is easily accessible from the keyboard application to be shared with the mental health professional. With this UUID, the mental health professional can register the patient on the web server through the patient management function of the data visualization tool.

The web platform consists of three software components, as shown in Figure 1: a web service, an inference engine with the classification model, and a web application. The service was implemented using the FastAPI framework [42]. It has an application programming interface (API), built in Python, that is responsible for receiving the data sent by virtual keyboards. A secure sockets layer (SSL) is the secure communication protocol used to transmit data between the virtual keyboard and the web service over an encrypted link. The existence of terms associated with suicide is checked [43] (see Table 1): if any suicide-related term appears in the sentence, the text is then sent to the inference engine; if the sentence has no suicide-related term, it is already considered negative for suicidal ideation. In this second case, texts are immediately discarded by the web service. Sentences received in the inference engine are processed and classified by a classification model (described in Section 2.3). After the classification process, texts are also destroyed. Therefore, there is no storage of texts in any part of the system, and mental health professionals only have access to classification results.

Finally, the classification results from both the web service (results obtained from sentences considered negative for suicidal ideation because they do not even have the suicide-related terms) and the inference engine (that is, predictions classified as positive or negative for suicidal ideation, depending on the classification model results) are sent to the web application, where they are stored in a database and displayed in dashboards to mental health professionals. The web application was developed using the Laravel framework [44]. This tool is also responsible for providing dashboards and managing the user accounts of professionals, who can define patients linked by UUIDs. Dashboards display individual results per patient and summarize results of all patients. In addition, the tool provides a summarization dashboard that can be configured according to the number of positive sentences for suicidal ideation, which can represent a risky situation.

Professionals only have access to data from their patients. Therefore, as texts are discarded after processing, only numerical and graphic information of those patients which the professional is responsible for providing health care to are presented by the system. Remarkably, the *Boamente* system does not provide any resource for mental health professionals to perform interventions. The system was developed to be a useful tool focused on the decision-making process for carrying out interventions in certain patients during face-to-face therapies or using other digital tools. Thus, the system is a tool for generating evidence to help the decision-making process of health professionals.

### 2.3. Identifying Suicidal Ideation

**Figure 2:**
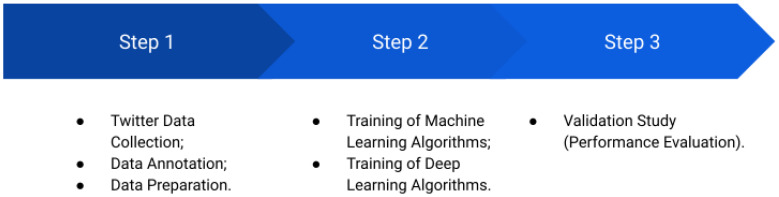
Methodology to find the best ML/DL model to be deployed in the inference engine.

The core of the *Boamente* system is its inference engine capable of classifying suicide-related texts. To build and identify the best ML/DL model to be deployed in it, we followed the methodology illustrated in Figure 2, which is the state-of-the-art process in sentiment analysis tasks [45]. In Step 1, we collected Twitter posts (non-clinical texts), sent them for annotation by psychologists as positive or negative for suicidal ideation, and performed the processes of cleaning and preparing the texts to be used as inputs in the training of the ML/DL algorithms (Step 2). Finally, in Step 3, we evaluated and compared the performance of the models to find the best model. These steps are detailed below.

#### 2.3.1. Data Collection and Annotation

We obtained non-clinical texts from tweets (user posts of the online social network Twitter). To find suicide-related tweets, we used the Twitter API to download tweets in a personalized way based on search terms associated with suicide [43] (Table 1). After different experiments to retrieve relevant texts, a total of 5699 tweets were collected in May 2021. Each downloaded tweet had various user-specific information (for example, user ID, timestamp, language, location, number of likes, etc.), but we kept only the post content (suicide-related texts) and discarded the additional data. Therefore, all texts were anonymized. The dataset produced in our study cannot be made publicly available due to Twitter’s developer agreement and policy [46], which restricts the redistribution of tweets to third parties. However, the dataset is available upon request from the corresponding author.

After data collection, three psychologists were invited to perform the data annotation, in which they individually labeled each tweet. To avoid bias in the annotation process, we selected psychologists with different psychological approaches, namely: cognitive behavioral theory, psychoanalytic theory, and humanistic theory. Professionals had to classify each tweet as negative for suicidal ideation (annotated as 0), or positive for suicidal ideation (annotated as 1).

Table 2 displays two examples of tweets labeled by psychologists. The texts were originally written in Portuguese and then translated to English by the authors. Therefore, some subtle changes in their meaning may have been introduced, in spite of the efforts to make the best possible translation. All tweets that had at least one divergence between psychologists (n = 1513) were excluded, resulting in a dataset with 4186 instances. Before the dataset was submitted to data preparation (Section 2.3.2), 398 duplicate tweets were excluded. The final dataset consists of 2691 instances labeled negative and 1097 labeled positive.

#### 2.3.2. Data Preparation

To prepare the data, the following procedures were applied to the texts [45,47]:

1. Text cleaning: removal of terms that are out of context, such as uniform resource locators (URLs), email addresses, symbols, and numbers;
2. Stop words removal: removal of words that do not contribute to the analysis (for example, “as”, “e”, “os”, “de”, “para”, “com”, “sem”, “foi”);
3. Tokenization: procedure responsible for separating texts into smaller units named tokens (that is, a sentence is divided into words);
4. Stemming: reduces inflection in words to their basic form. For example, the words “gato” (male cat in PT-BR), “gata” (female cat in PT-BR), “gatos” (male cats in PT-BR), and “gatas” (female cats in PT-BR) would reduce to “cat” (the stem);
5. Term Frequency–Inverse Document Frequency (TF-IDF): a statistical measure that evaluates how relevant a word is to a sentence in a collection of sentences, which is very useful for scoring words.

**Figure 3:**
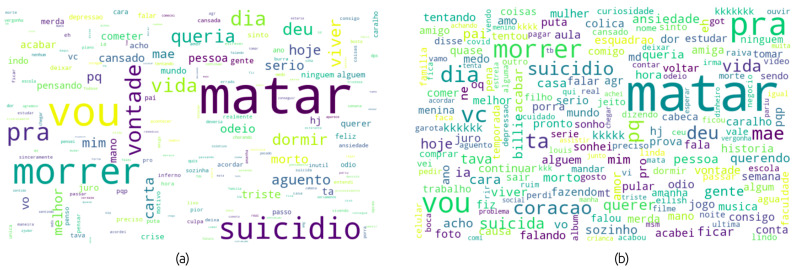
Word clouds containing terms after text cleaning and stop words removal for (**a**) positive class and (**b**) negative class.

Importantly, only procedure 1 was performed to train DL algorithms, and all of them were used in the training of ML algorithms. Figure 3 presents word clouds containing terms in PT-BR after applying procedures 1 and 2, explained above, for positive and negative classes.

**Figure 4:**
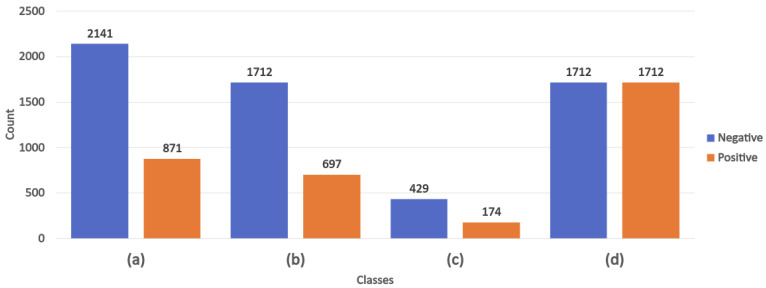
Number of instances labeled as negative and positive: (**a**) without data balancing (original dataset); (**b**) with 80% used for training and validation; (**c**) with 20% used for testing; and (**d**) with 80% used for training after applying SMOTE.

A dataset is considered to be imbalanced when the classification categories are not equally represented—in other words, when one class has more instances than another (or others). In the textual dataset used in our study, the major class is “negative” (Figure 4a). We first split the dataset, with 80% used for training and validation (Figure 4b), and 20% used for testing (Figure 4c). Then, we had to balance the two classes (Figure 4d) for model performance optimization purposes. To do this, we used the synthetic minority over-sampling technique (SMOTE), which generates new instances (synthetic observations) from existing minority cases [48].

#### 2.3.3. Training of ML/DL Algorithms

To take advantage of better computing power, prepared textual data were submitted as inputs for training ML algorithms using Google Colaboratory [49] and DL algorithms using Kaggle [50] in Python. In Google Colaboratory, we used a cloud computational environment with the following specifications: CPU Intel Xeon 2.20 GHz with 2 cores, no use of GPU, and 12 GB RAM; in Kaggle, the specifications were: Intel Xeon 2.20 GHz with 4 cores, GPU Nvidia Tesla P100 with 16 GB, and 16 GB RAM. We utilized the Scikit-learn library [51] to train the ML algorithms. We conducted experiments with several ML algorithm implementations provided by Scikit-learn to define the classifiers to be explored for comparison purposes with the DL models. With this aim, we trained different classifiers and adjusted their hyperparameters (that is, fine-tuning) to optimize the performance of the models with the grid search technique. Such a technique exhaustively tests different combinations of hyperparameters to find the one with the best performance. In the end, the best performance results, according to the metrics described in Section 2.3.4, were obtained by models using the following two algorithms: random forest classifier and an ensemble of decision trees, called extra trees classifier.

We also trained the DL algorithms to find improved performance in the suicide-related text classification task with our dataset. In fact, we fine-tuned three different versions of the pretrained BERT model for Portuguese [52]—Multilingual BERT (base) [39,53] and BERTimbau [54,55,56] (base and large)—all of them in the case-sensitive form. BERT is a pretrained language model that uses bidirectional transformers and that can be fine-tuned with one additional output layer to create DL models. It has achieved state-of-the-art performance in a wide range of NLP tasks. BERTimbau is a specialized version of BERT for PT-BR, which was trained using data from the Brazilian Portuguese Web as Corpus (brWaC) [57,58], a large and diverse corpus of web pages in PT-BR.

As BERT is an attention model that considers the context of each word in the sentence during training, only the text cleaning (procedure 1 in Section 2.3.2) was performed for data preparation. The development of BERT-based models was also conducted using Google Colaboratory in Python, supported by TensorFlow [59] and PyTorch [60] libraries. Finally, a total of five models were compared: two ML models and three DL models.

#### 2.3.4. Validation Study

**Figure 5:**
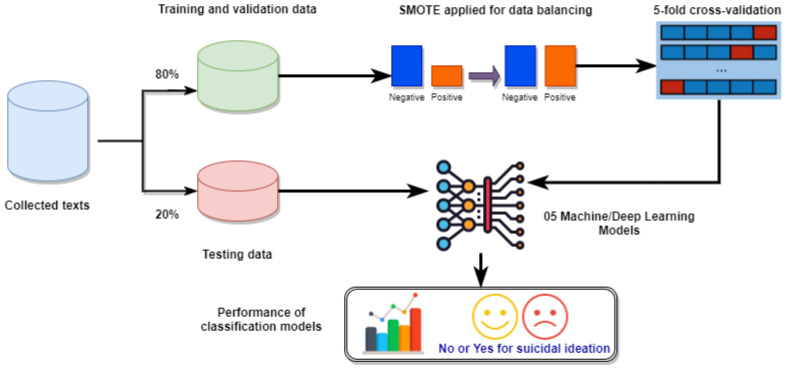
Cross-validation applied for evaluating the performance of all models.

Figure 5 illustrates the cross-validation process of the models used in this work. The dataset was divided into 80% for training/validation and 20% for testing. The SMOTE technique was applied to balance the positive and negative classes in the training and validation dataset. Balanced data were used to build each of the five models through 5-fold cross-validation [61,62]. After the training phase, the five models were applied to the testing dataset with performance metrics based on the confusion matrix, as shown in Table 3, with values described as follows.

- True positive (TP): correct prediction of positive value;
- True negative (TN): correct prediction of negative value;
- False positive (FP): wrong prediction of negative value (a contradiction);
- False negative (FN): wrong prediction of positive value (a contradiction).

We analyzed the following well-known performance metrics [63]: accuracy (Equation (1)), precision (Equation (2)), recall (Equation (3)), and F-score (Equation (4)). We also analyzed receiver operation characteristic (ROC) curves and the area under the ROC curve (AUC), which can display trade-offs between sensitivity (Equation (3)) and specificity (Equation (5)) outcomes.

(1) $$Accuracy=\frac{TP+TN}{TP+TN+FP+FN}$$

(2) $$Precision=\frac{TP}{TP+FP}$$

(3) $$Recall=\frac{TP}{TP+FN}=Sensitivity$$

(4) $$F-score=\frac{2*(Precision*Recall)}{Precision+Recall}$$

(5) $$Specificity=\frac{TN}{FP+TN}=1-\frac{FP}{FP+TN}$$

The recall metric is the number of true positives out of the actual positive texts. Although we analyzed several metrics, we prioritized using recall to define our best model because it considers the true-positive rate. Such a rate is important when identifying suicidal ideation, as false negatives matter, more than false positives, to the process of detecting a risk of suicidality. After defining the best model, we created and analyzed a sensitivity/specificity report through ROC curve and AUC to know the discriminative ability of the model.

## 3. Results

### 3.1. *Boamente* Tool

**Figure 6:**
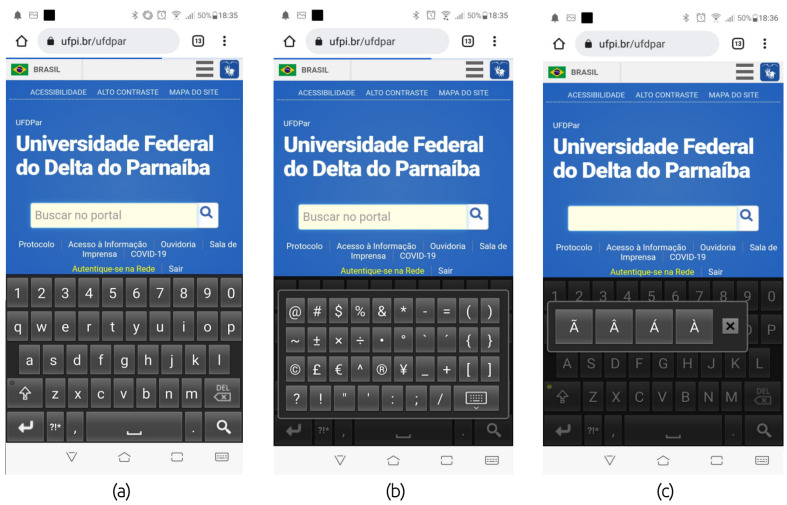
Screenshots of the *Boamente* virtual keyboard.

Figure 6 presents screenshots of the *Boamente* virtual keyboard in the dark theme: (a) the keyboard used to write a URL in a browser application; (b) the main special characters available in the keyboard; and (c) accentuation of the Portuguese language. The keyboard is very similar to the default Android keyboard, and it has dark and light themes.

**Figure 7:**
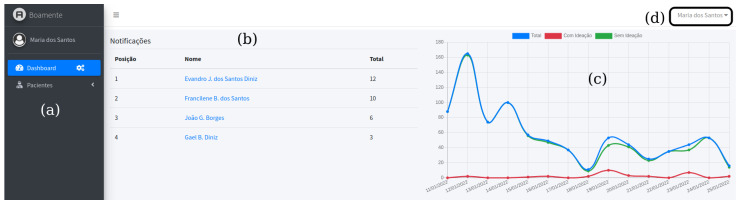
Dashboards on the *Boamente* web application home screen.

The *Boamente* web application has an access control list (ACL) with role-based permissions for two types of users: the system administrator, who has permission to manage users of mental health professionals; and professionals, who register and manage patient records and access the dashboards of their patients. Figure 7 depicts the home screen accessible to professionals, which presents: (a) the system menu; (b) a dashboard with a ranking of all patients who had a specific number of texts positive for suicidal ideation within a time interval—the number of texts and the time interval is defined by the professional; (c) a line chart that shows the sum of texts from all patients (blue line), with the red line representing positive predictions, and the green line corresponding texts without suicidal ideation; and (d) the name of the mental health professional logged into the system. From this home screen, mental health professionals have a broad view of their patients and, if necessary, can access patient-specific dashboards.

**Figure 8:**
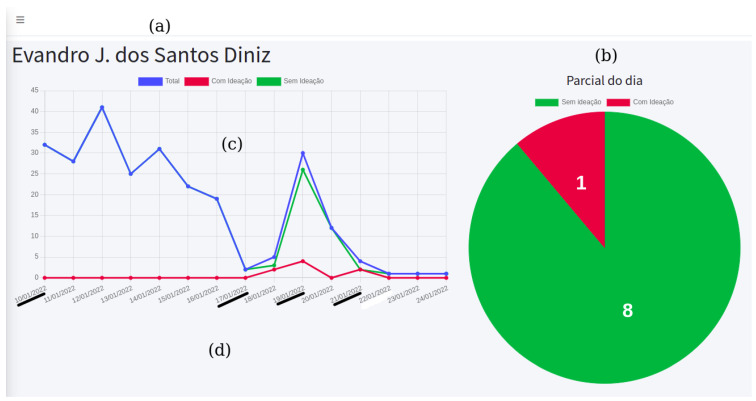
Patient-specific dashboards on the *Boamente* web application.

When accessing a patient’s information, the professional can see two dashboards, as illustrated in Figure 8: (a) the name of the patient is displayed; (b) a pie chart that displays the number of partial predictions for the current day, with information for positive (red part) and negative (green part) classifications; (c) a line chart in which predictions are displayed according to the period of time determined by the professional, with similar colors (blue line for the total number, red for positive predictions, and green for negative ones); and, in Figure 8d, the evolution of the classification results for the selected patient are shown, highlighting four days underlined. On 10/01/2022 (date format dd/mm/yyyy, which is normally used in Brazil), there was no positive prediction recorded. From 17/01/2022, after a drop in records, the patient typed sentences classified as positive for suicidal ideation, extending until 19/01/2022 and repeating on 21/01/2022. From that day, the patient barely sent messages for three days, compared to the beginning of the selected interval, then returned to communication only on 25/01/2022 (Figure 8b presents a daily partial with nine texts). Both Figure 7 and Figure 8 are illustrative examples used to exemplify different scenarios. As a support tool that allows analysis performed on dashboards, mental health professionals can decide to carry out appropriate interventions during face-to-face therapies or using other digital methods/tools whenever they deem them necessary.

### 3.2. Model Performance

First, several classifiers provided by Scikit-learn were trained. The runtime required to train them was insignificant. We present the five models that achieved the best performance results in Table 4. All of them had their hyperparameters fine-tuned with the grid search technique, which allowed us to select the two outstanding ML models. However, as we can see, the performance results of these five models were very close. The two models that stood out, which we selected for comparison purposes with the DL models, were the random forest classifier and the extra trees classifier, which achieved a recall of 0.895 and 0.877, respectively.

**Figure 9:**
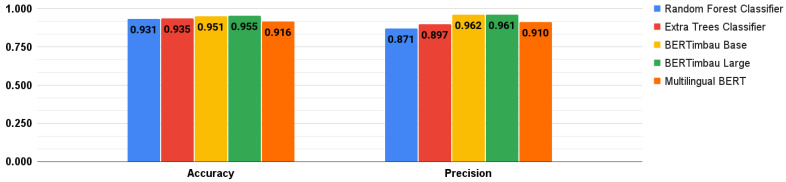
Best results for accuracy and precision metrics.

**Figure 10:**
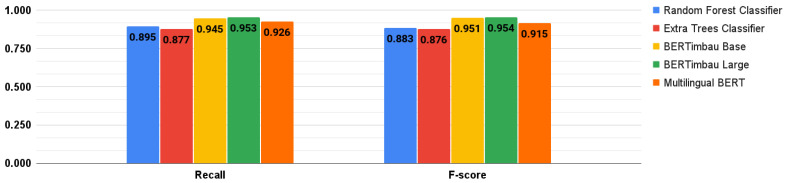
Best results for recall and F-score metrics.

The three BERT-based models were also fine-tuned. To do so, we trained them following the suggestions in [39], with all combinations of hyperparameters: batch sizes of 16 and 32; Adam learning rate of 5 × 10^−5^, 3 × 10^−5^, and 2 × 10^−5^, and number of epochs (2, 3, and 4) [64]. Therefore, eighteen training sessions were performed for each model. Runtimes to train these models in each session varied according to the hyperparameters used. The average session times for each model were: Multilingual BERT: 7 min and 15 s; BERTimbau Base: 6 min and 31 s; and BERTimbau Large: 22 min and 55 s. Figure 9 and Figure 10 show the best performance results achieved by the DL/ML models, both fine-tuned. The best BERT-based model was BERTimbau Large, and its best combination of hyperparameters (batch size of 16; Adam learning rate of 2 × 10^−5^; and 4 epochs) returned the following results: accuracy of 0.955; precision of 0.961; recall of 0.953; and F-score of 0.954.

**Figure 11:**
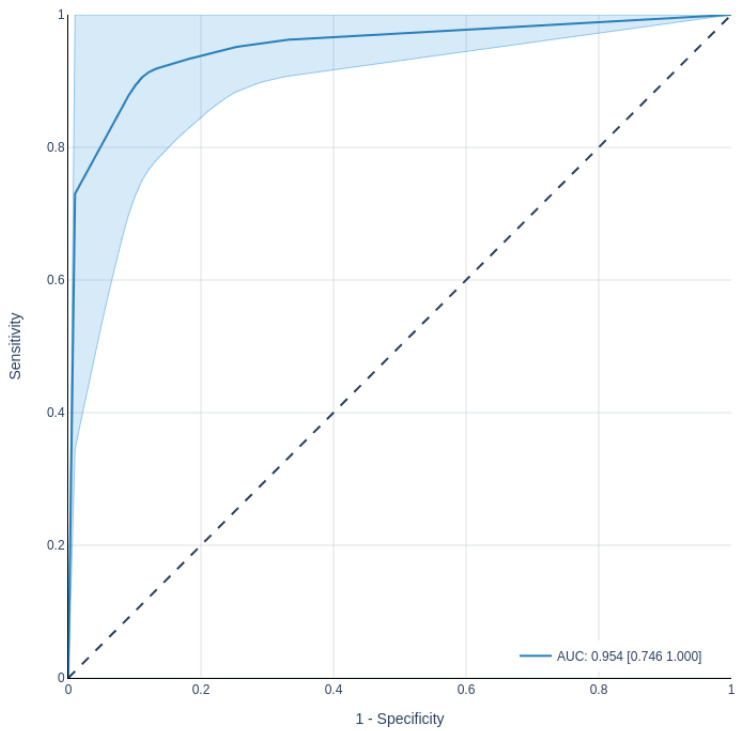
ROC curve with confidence interval of the BERTimbau Large model.

The ROC curve presents the performance of a classifier, especially in binary problems (in the case of this study, the classes are negative and positive for suicidal ideation), through a visual representation of the balance between the true-positive rate (sensitivity) and the false-positive rate (1—specificity), varying the threshold (for example, the cutoff point for the estimated probability) [65]. The area under the ROC curve can be used as a single measure, but with relevant probabilistic interpretation, independent of the classification threshold, and with fewer deficiencies than the classification error rate [66]. It ranges from 0 to 1, where AUC = 0 represents totally incorrect classifications, and AUC = 1 represents totally correct ones. It is desirable to have a result that is both sensitive and specific. Figure 11 presents the ROC curve with 95% confidence regions of our classifier (BERTimbau Large model). In the graph, we can see the average AUC = 0.954 (MIN: 0.746; MAX: 1.0) that considers the five folds of cross-validation when classifying instances of the testing dataset. This AUC demonstrates the discriminating power of sensitivity and specificity, indicating that the model can achieve low false-positive and false-negative rates.

## 4. Discussion

### 4.1. Contributions and Applicability of the *Boamente*

An increasing number of mobile solutions have been proposed in the literature for digital phenotyping [13]. Moreover, DL models have been intensively explored in combination with data from social media to detect suicidal ideation [67]. However, two research gaps remain: (1) there is a lack of solutions that deploy the models developed for suicidal ideation detection (different models have been developed but not deployed in solutions used in clinical settings) [23]; and (2) the passive monitoring of textual patterns is a too-little explored method in digital phenotyping studies [12,13]. Our study aimed to fill such gaps by proposing a digital phenotyping solution that monitors suicidal ideation from texts typed by users in smartphones.

Specifically, we propose *Boamente* to allow health professionals to monitor the suicidal ideation of their patients. To develop the *Boamente* model, we first collected a dataset with non-clinical texts in Brazilian Portuguese obtained from Twitter. Next, tweets were classified by three psychologists as positive or negative for suicidal ideation. The dataset was then preprocessed and used as the input to develop machine learning and deep learning models. Therefore, we provided the model with different sentences: texts containing actually suicidal ideations from people actually considering taking their lives, and texts containing terms related to suicide, but used in different contexts (for example, irony, jokes, sarcasm, or meaningless sentences). Finally, we deployed the model with the best performance result in the *Boamente* system to enable the smart monitoring of suicidal ideation.

Our proposed solution consists of complementary components. It has a virtual keyboard application to passively collect user texts and send them to a web platform. The keyboard does not provide the autocomplete function (a feature to predict the rest of a word while a user is typing), which forces users to write texts by themselves. In addition, it does not capture texts from the clipboard, which ensures that texts sent to the web platform are created/typed by users. Different from solutions for clinical text classification [68], the *Boamente* web platform classifies non-clinical texts according to the presence or absence of suicidal ideation and presents results in dashboards to mental health professionals.

NLP-based systems focused on detecting suicidal ideation can determine whether an individual has suicidal ideation or thoughts by analyzing textual content written by them [22]. Therefore, *Boamente* can be categorized as a system for suicidal ideation detection, which is focused on the early detection and prevention of suicide attempts. Importantly, *Boamente* is unable to predict future risk to suicidal ideation, such as the work proposed by Roy et al. [69]. Otherwise, it records current data and enables the analysis of historical data through several charts displayed in dashboards.

*Boamente* was mainly developed to be used by patients undergoing treatment with mental health professionals. However, we understand that the reasons why people commit suicide are complicated. For example, depressive individuals are extremely likely to commit suicide, but many people without depression can also have suicidal thoughts [70]. In addition, youth and teenagers may disclose risk factors for suicide on social media that they do not disclose to mental health professionals [71]. Therefore, the use of the *Boamente* keyboard should be a recommendation of specialized professionals to patients with any evidence of suicide risk. For instance, professionals may wish to monitor not only patients with severe mental disorders who have indication of suicidality, but also patients with milder symptoms.

### 4.2. Model Performance Analysis

We trained several ML and DL models for text classification. Two ML models (random forest classifier and extra trees classifier) and three DL models (Multilingual BERT and two variations of BERTimbau) were selected for comparison. All models were fine-tuned, in which different combinations of hyperparameters were tested, and the best model (BERTimbau Large) was selected by considering the recall metric. We expected that one of the pretrained BERTimbau models would be selected as the best one, since they are considered state-of-the-art deep learning models for text classification in many tasks when using Brazilian Portuguese [54].

**Figure 12:**
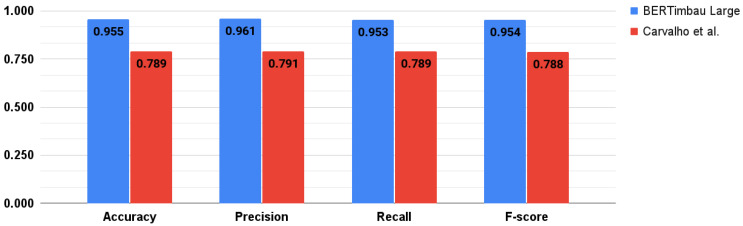
Comparison of our best model with the best one developed in Carvalho et al. [38].

As described in Section 1, only the work by Carvalho et al. [38] was considered similar to our study, and we considered the models developed by them as our initial baseline. As presented in Figure 12, our best model (recall of 0.953) outperformed the best results presented in [38] (recall of 0.789) for binary classification. In fact, all our five compared models performed better.

### 4.3. Limitations and Future Work

Passive health data has the potential to revolutionize healthcare. Nevertheless, due to ethical concerns [72], there is still a lack of consensus regarding the use of this type of data. Textual data gathered by the *Boamente* keyboard are not controlled by the user and, therefore, the keyboard has no privacy control definitions available. Although all texts sent to the server side are discarded after processing, the user can stop sending texts only when switching keyboard applications. This is a limitation of the current version of our proposed solution. Therefore, future work will develop privacy controls to be made available on the keyboard. Moreover, we are planning to develop a solution to register when the user switches between different keyboards installed on the smartphone. This additional resource will identify when the user is purposely disabling the use of the keyboard, which may represent non-adherence to professional monitoring. Another limitation of our proposed solution is not being able to detect typos, which could possibly lead to text misclassifications; thus, we plan to develop functions such as autocomplete and spell checker.

Future plans also include developing ML/DL models for the early detection of other mental disorders (for example, depression, anxiety), which will improve the potentialities of the *Boamente* tool. In parallel, we intend to investigate the use of transfer learning in classification tasks of texts related to mental health. Moreover, since evidence in decision support tools is required to be explainable, we would also like to dedicate efforts to provide transparency to the *Boamente* system, which will be useful to mental health professionals. An explanation of how the DL model generates their outputs, without compromising data privacy, is desirable [73]. Finally, further investigation is aimed at conducting experimental studies with professionals and patients, such as a usability and user-experience evaluation and, subsequently, a clinical trial.

## 5. Conclusions

In this paper, the *Boamente* tool was proposed to monitor the suicidal ideation of users. Our mobile mental health system is based on the concept of digital phenotyping by using texts passively collected from a virtual keyboard. It has a DL model that classifies user-generated texts according to the existence of suicidal ideation. Our solution was co-designed by mental health professionals to be used in clinical settings. To enable it for this purpose, classification results are stored in a database to be consulted by mental health professionals via dashboards, which can allow them to monitor their patients. The performance evaluation results of the model selected to be deployed in the system (BERTimbau Large) were demonstrated to be promising. Therefore, the *Boamente* tool can be effective for identifying suicidal ideations from non-clinical texts, which enables it to be experimented with in studies with professionals and their patients.

## Figures and Tables

**Table 1:** Suicide-related terms and expressions.

Portuguese Language (PT-BR)	English Language
suicida	suicidal
suicídio	suicide
me matar	kill myself
meu bilhete suicida	my suicide note
minha carta suicida	my suicide letter
acabar com a minha vida	end my life
nunca acordar	never wake up
não consigo continuar	can’t go on
não vale a pena viver	not worth living
pronto para pular	ready to jump
dormir pra sempre	sleep forever
quero morrer	want to die
estar morto	be dead
melhor sem mim	better off without me
melhor morto	better of dead
plano de suicídio	suicide plan
pacto de suicídio	suicide pact
cansado de viver	tired of living
não quero estar aqui	don’t want to be here
morrer sozinho	die alone
ir dormir pra sempre	go to sleep forever

**Table 2:** Examples of tweets labeled by psychologists.

Class	Tweet (PT-BR)	Tweet (English)
Negative	meu sonho é dormir pra sempre mas quem dorme pra sempre eh quem morre mas eu não quero morrer só quero dormir pra sempre msm.	my dream is to sleep forever, but the one who sleeps forever is the one who dies, but I don’t want to die, I just want to sleep much.
Positive	daí você mistura um monte de remédios esperando sei lá dormir pra sempre e acorda já no dia seguinte só com uma dor no estômago absurda acordada triste com dor no estômago mais azarada que eu.	then you mix a bunch of meds hoping, I don’t know, to sleep forever and wake up the next day only with an absurd stomachache, I’m awake sad, and my stomach hurts, more unlucky than me.

**Table 3:** Confusion matrix.

		Actual Values		
		Positive	Negative	Total
Predicted Values	Positive	*True Positive*	*False Positive*	TP+FP
	Negative	*False Negative*	*True Negative*	FN+TN
	Total	TP+FN	FP+TN	

**Table 4:** Performance of the machine learning models.

Algorithms/Metrics	Accuracy	Precision	Recall	F-Score
SVC	0.902	0.825	0.840	0.832
Extra trees classifier	0.935	0.897	**0.877**	0.876
Random forest classifier	0.931	0.871	**0.895**	0.883
Gradient boosting classifier	0.866	0.723	0.870	0.789
MLP classifier	0.873	0.745	0.855	0.796

## Data Availability

The data presented in this study are available on request from the corresponding author. The data are not publicly available due to containing information that could compromise the privacy of their owners.

## Abbreviations

The following abbreviations are used in this manuscript:

ACL	Access Control List
AUC	Area Under Curve
BERT	Bidirectional Encoder Representations from Transformers
BrWaC	Brazilian Portuguese Web as Corpus
DL	Deep Learning
FN	False Negative
FP	False Positive
ICT	Information and Communication Technologies
IDE	Integrated Development Environment
ML	Machine Learning
NLP	Natural Language Processing
PT-BR	Brazilian Portuguese
ROC	Receiver Operation Characteristic
SSL	Secure Sockets Layer
SVC	C-Support Vector Classification
TF-IDF	Term Frequency–Inverse Document Frequency
TN	True Negative
TP	True Positive
URL	Uniform Resource Locator
UUID	Universally Unique Identifier
WHO	World Health Organization
